# 

**Published:** 1920-03-06

**Authors:** 


### Matrons' and Sisters' Appointments.

Royal Albert Edward Infirmary, Wigan.—Miss S. A. Eddy, ward sister, has been promoted to be night sister. She was trained at the Royal Infirmary, Sheffield.

Royal Hospital for Sick Children, Edinburgh.—Miss Maud Cobley has been appointed night sister. She was trained at Ashton-under-Lyne District Infirmary, where she was later sister, and she has been night sister at the Victoria Hospital for Children, Hull, ward and night sister at Oldham Infirmary, and sister of the medical and surgical wards at the County Hospital, Lincoln.

Essex County Hospital, Colchester.—Miss Mabel Halls has been appointed sister of the women's surgical ward. She was trained at the above-named institution, where she afterwards filled the post of staff nurse. Miss Halls holds the certificate of the Central Midwives Board.

Royal Albert Edward Infirmary, Wigan.—Miss Beatrice Barford has been appointed housekeeping sister. She was trained at Hackney Infirmary, where she was later staff nurse, ward and theatre sister, and night superintendent, and she has been night superintendent, theatre sister, and temporary assistant matron at Torbay Hospital, Torquay. Miss Barford holds the certificate of the Central Midwives Board.

Leeds Maternity Hospital.—Sister Harriet Heighway has been appointed assistant night sister. She was trained at the Royal Southern Hospital, Liverpool, and at the Liverpool Maternity Hospital, and has been sister at Rochdale Infirmary.

Home for Ailing Babies, Woolwich.—Mrs. C. Williams has been appointed sister. She was trained at the Croydon Union Infirmary, has been staff nurse at Queen Mary's Hospital, Carshalton, and ward sister at the Northern Convalescent Fever Hospital, Winchmore Hill.

Lady Minto's Indian Nursing Association.—The following have been appointed sisters: Miss Frances Margaret Wallis and Miss Elsie Mabel Osborne, trained at the Great Northern Central Hospital; Miss A. E. Roberts, trained at Bristol Royal Infirmary; Miss Florence Alice Wild, trained at Sheffield Royal Infirmary; and Miss Lilian Jane Walker, trained at Withington Hospitals, Manchester.

Union Infirmary, Leigh.—Miss Alice H. Scott has been appointed night charge nurse. She was trained at Brownlow Hill Infirmary, Liverpool, and has since been sister at the Royal National Hospital, Ventnor, at the Royal Naval Hospital, Dungavel, N.B., night superintendent at Middleton-in-Wharfedale Sanatorium, and senior sister and deputy matron of the Victoria Hospital, Accrington.

Halifax, St. Luke's Hospital.—Miss Minnie Fuller has been appointed sister. She was trained at Manchester Royal Infirmary, where she was later sister. She has also been sister at the Devonport Hospital, Plymouth.

### The College of Nursing (Limited by Guarantee).

### THE COLLEGE BADGE.

We are informed by the College of Nursing that it may be some weeks before members will receive their badges. The orders, which are coming to the office in large numbers, are being taken in rotation, and the badges will be despatched as soon as possible. Suggestions have been made that the name of the owner be engraved on the back of the badge, but it is pointed out that, in order to protect the badge from use by impostors, it has been decided to record the registration number only. Should any impostor obtain possession of a College badge, it would be perfectly easy for her to assert ownership by assuming the name engraved on the back, but it is quite unlikely that an impostor would know the name corresponding to the number. For purposes of evidence the name can always be verified at headquarters.

### Forthcoming Events.

**National Hospital for the Relief and Cure of the Paralysed and Epileptic.**
(Albany Memorial), Queen Square, W.C.

Annual General Meeting at the hospital, Tuesday, March 23, 1920, 4.30 p.m.

Seamen's Hospital Society.—Annual Court of Governors at Queen's Hall, Langham Place, W., Thursday, March 11, 3.45 p.m., under the presidency of Admiral of the Fleet the Right Hon. Earl Beatty, G.C.B., G.C.V.O., supported by the Right Hon. A. J. Balfour, O.M., M.P., the Right Hon. Dr. Addison, M.P., Admiral of the Fleet Sir Henry Jackson, G.C.B., F.R.S., and others.

The Chadwick Public Lectures will be given on Mondays, March 8, 15, and 22, at 5.15 p.m., in the Lecture-room of the Royal Society of Arts, John Street, Adelphi, W.C. 2. The lectures, which are by General Sir John Goodwin, K.C.B., C.M.G., D.S.O., will be on "Military Hygiene in Peace and War."

### The Book Market.

Longmans, Green and Co.: "The Physiology of Muscular Exercises." By F. A. Bainbridge, M.A., M.D. 10s. 6d. net.

Baillière, Tindall and Cox: "Child Welfare." By Sim Wallace, M.D. 5s. net.

J. and A. Churchill: "Venereal Disease: its Prevention, Symptoms, and Treatment." By Wansey Bayly. "Minor Surgery Bandaging." By Gwynne Williams, M.S., F.R.C.S. 10s. 6d. net.

E. S. Livingstone: "Toxicology" (2nd edition); "Physics" (Part 1); Zoology" (Part 1). 1s. 6d. net each. "Medical and D.P.H. Examination Papers." 3s. net.

Andrew Melrose, Ltd.: "Thryrea and other Sonnets." By John Ferguson. Sixth edition. 1s. net.

Oxford Medical Publications: "Surgical Operations: A Text-Book for Nurses." By E. W. Hey Groves, M.D. 21s. net.

Crosby Lockwood and Son: "Mechanical Dentistry." By Charles Hunter. 5s. net.

Constable and Co., Ltd.: "All About Baby." By A. R. Lenman. 1s. 6d. net.

Williams and Norgate: "Engines of the Human Body." By Prof. Arthur Keith, M.D., &c. 12s. 6d. net.

John Lane, The Bodley Head: "Domus Dolores." By W. Compton Leith. 7s. 6d.

Adlard and Son, Ltd.: "Transactions of the National Association for the Prevention of Tuberculosis." Seventh Annual Conference. 10s., post free. "Handbook of Tuberculosis Schemes. Vol. I. England and Wales. 5s.

### Recent Official Publications.

(To be obtained from H.M. Stationery Office, Imperial House, Kingsway, W.C. 2. Prices include postage.)

Medical Research Committee: Special Reports, No. 41, An Investigation into the Ultimate Results of the Treatment of Syphilis with Arsenical Compounds. A Clinical Study of the Toxic Reactions which follow the Intravenous Administration of "914." 2s. 1½d. No. 42, A Study of the Serological Races of the Flexner Group of Dysentery Bacilli. 2s. 1½d. No. 43, Albuminaria and War Nephritis Among British Troops in France. 2s. 8½d. No. 39, Anærobic Infections of Wounds and the Bacteriological and Serological Problems Arising Therefrom. 6s. 3½d. Statistical Report No. 5, Statistical Reports from the British Forces in France on Penetrating Wounds of the Chest. 1s. 7½d. Special Report Series, No. 45, Unsuspected Involvement of the Central Nervous System in Syphilis. 1s. 1½d. No. 44, Reports of the Special Committee on Manufacture, Biological Testing, and Clinical Administration of Salvarsan and its Substitutes. 1s. 2d.

### Question for March.

### WIDENING THE AREA OF HOSPITAL SUPPORTERS.

Elaborate a plan for obtaining the financial assistance of members of the middle classes who do not at present contribute to hospital funds and who cannot afford to become annual subscribers. Outline methods of working, without involving an increase of staff.

**(A minimum payment of 10s. 6d. will be made for each published answer.)**

### RULES.

The following rules must be observed:—

1. Contributions must be written on one side of the paper. Conciseness and terseness are desirable features. The MS. must bear the name and address of the sender and be accompanied by coupon to be cut from the back cover (inside page) of the current issue of THE HOSPITAL. A pseudonym must be chosen if the name is not to be published.

2. Contributions must be addressed to the Editor of THE HOSPITAL Institutional Worker Supplement, 28 & 29 Southampton Street, Strand, London, W.C. 2, should reach him before the end of the current month, and be marked in the left-hand corner "Facts and Figures."

### QUESTIONS INVITED.

IN connection with our Question Box, we offer every month five shillings each for the best questions which are sent in for consideration. The questions may be on any institutional subject and concern any department, from the secretary's to the porter's, or the matron's to the domestic staff; the one essential is that they must be practical and deal with points that have a definite relation to hospital or institutional administration, upkeep, finance, or management. Points relating to artificers' work, housekeeping, the laundry, out-patients, and other practical matters will be welcome. It is hoped that thus, when difficult or doubtful points arise in the course of their work, institutional workers will be encouraged to put them in the form of a question and send them to the Editor, THE HOSPITAL Bureau of Information, 28 & 29 Southampton Street, Strand, London, W.C. 2, marked "Question Box."

The best questions will be published in due course, and our readers will be given the opportunity of answering them. Thus all institutional workers may be able to co-operate with the view of helping the work and smoothing the difficulties of each other. In short, we want the Question Box of the *Institutional Worker* to be freely used by every worker habitually.

### ENQUIRIES AND ANSWERS.

### *EMPLOYMENT AND TRAINING.*

### Hostel for Queen's Nurse in London.

IF you are to be in London just for a short time on leave, write to the Superintendent of Queen Mary's Hostel for Nurses, 194 Queen's Gate, London, S.W., and see if she can give you accommodation. The hospitality at this hostel is quite free.—QUEEN'S NURSE.

### Post-Certificate School for Midwives.

A POST-CERTIFICATE school such as you suggest has been opened at 77 Southampton Street, Camberwell, London, S.E. 5. A two months' course is given. Write to the Sister-in-Charge for a syllabus and all particulars.—COUNTRY MIDWIFE.

### Training School for Resident Masseuse.

THE following institutions have massage schools attached and receive resident pupils:—Bristol Royal Infirmary, and the Liverpool Royal Infirmary; the National Hospital and University College Hospital School of Massage and Electrical Treatment, 29 and 30 Queen Square, W.C. 1, arrange board and residence for women students. Write to the Matrons of the first two named institutions and the Secretary of the last named institution. —P. O.

### Nursing in India.

IF you would care for a Government appointment in India, write to the Medical Department, Queen Alexandra's Military Nursing Service for India, India Office, Whitehall, London, S.W. If you prefer private nursing write to Miss Ray, R.R.C., 54 Ashburnham Mansions, Chelsea, S.W. 10, for particulars of Lady Minto's Indian Nursing Service.—SISTER A.

### Homœopathic Hospital.

SUITABLE applicants are received at the London Homœopathic Hospital, Great Ormond Street, London, W.C., at the age of twenty-two for four years' training. There is a private nursing staff attached to the Hospital, and nurses who have completed their training are eligible for appointment upon it, and can in this case receive training in monthly nursing and midwifery. No premium is required. A salary is given from the first year.—K. A. C.

### Hospitals in Melbourne.

THE following are the only three large hospitals we know of in Melbourne:—The Alfred Hospital, Melbourne, beds 180; Melbourne Hospital, Lonsdale Street, beds 400; Children's Hospital, Pelham Street, Carlton, Melbourne, Victoria, beds 180. We suggest that you write to the matron of each institution, who will no doubt give you the information you require.—COLONIAL.

### *MISCELLANEOUS.*

### Membership of British Hospitals Association.

You should write to the Honorary Secretary, The British Hospitals Association, 1 Central Buildings, Tothill Street, Westminster, S.W., asking for rules and whether your hospital is eligible to join.—W. M.

### Useful Book for Surgical Nurses.

A MOST useful textbook for surgical nurses has recently been published upon "Surgical Operations." The book is written by E. W. Hey Groves, M.D., B.Sc., M.S., F.R.C.S., and is published by the Waverley Book Co., Ltd., 96 Farringdon Street, London, E.C. 4. The book begins with the reason why the operation is performed and continues by naming the instruments required, describes the steps of the operations, and concludes by giving an account of the special points to be observed in the after-treatment of each condition. The price of the book is 21s.; it is possible, however, to purchase the book by monthly payment.—THEATRE STAFF NURSE.

### Best Type of Urinal.

OUR own experience is that the best type of urinal becomes defective sooner or later, for the rubber of which they are made perishes. The type of urinal with inflated rubber pad at its contact with the body is the best, and if this is well fitted no leaking can take place until through wear and the action of the urine it breaks down. Probably your best course is to have two urinals, using them alternate days; thus one can be thoroughly cleansed whilst the other is in use. Two used in this way would last more than double the time of one in constant use. If you write to the Hospitals and General Contracts Company, Mortimer Street, W. 1, they will supply you with a urinal of this type. The price is 36s. Mr. Montague, of Bond Street, W., is also an expert in urinary apparatus.—B.

### A New Diathermy Attachment.

IF you have *x*-ray plant you can save a very considerable cost of a diathermic apparatus, which now amounts to about £80, by purchasing the "Victory" diathermy attachment, which has been recently placed on the market by the Medical Supply Association, Gray's Inn Road, London, W.C., the price of which is under £30. This piece of apparatus can be readily worked from an *x*-ray coil; it will produce diathermy current of the highest intensity required in practice, and is, in every way a thoroughly reliable instrument.—ELECTRICITY.

### Opaque Meal.

PROBABLY the most useful and popular form of opaque or shadow meal for use in *x*-ray examination of the alimentary canal is "Umbrose," prepared by Messrs. Allen and Hanbury, Ltd. This is a tabulum of cocoa, arrowroot, and desiccated milk, with 75 per cent. barium sulphate.—X-RAY.

